# Supplementary material for: Antigen-agnostic microfluidics-based circulating tumor cell enrichment and downstream molecular characterization
Source: PLoS One. 2020 Oct 23;15(10):e0241123. doi: 10.1371/journal.pone.0241123 (PMC7584183; doi:10.1371/journal.pone.0241123)
Supplement: S1 File — (PPTX) [file pone.0241123.s001.pptx]

## Slide 1
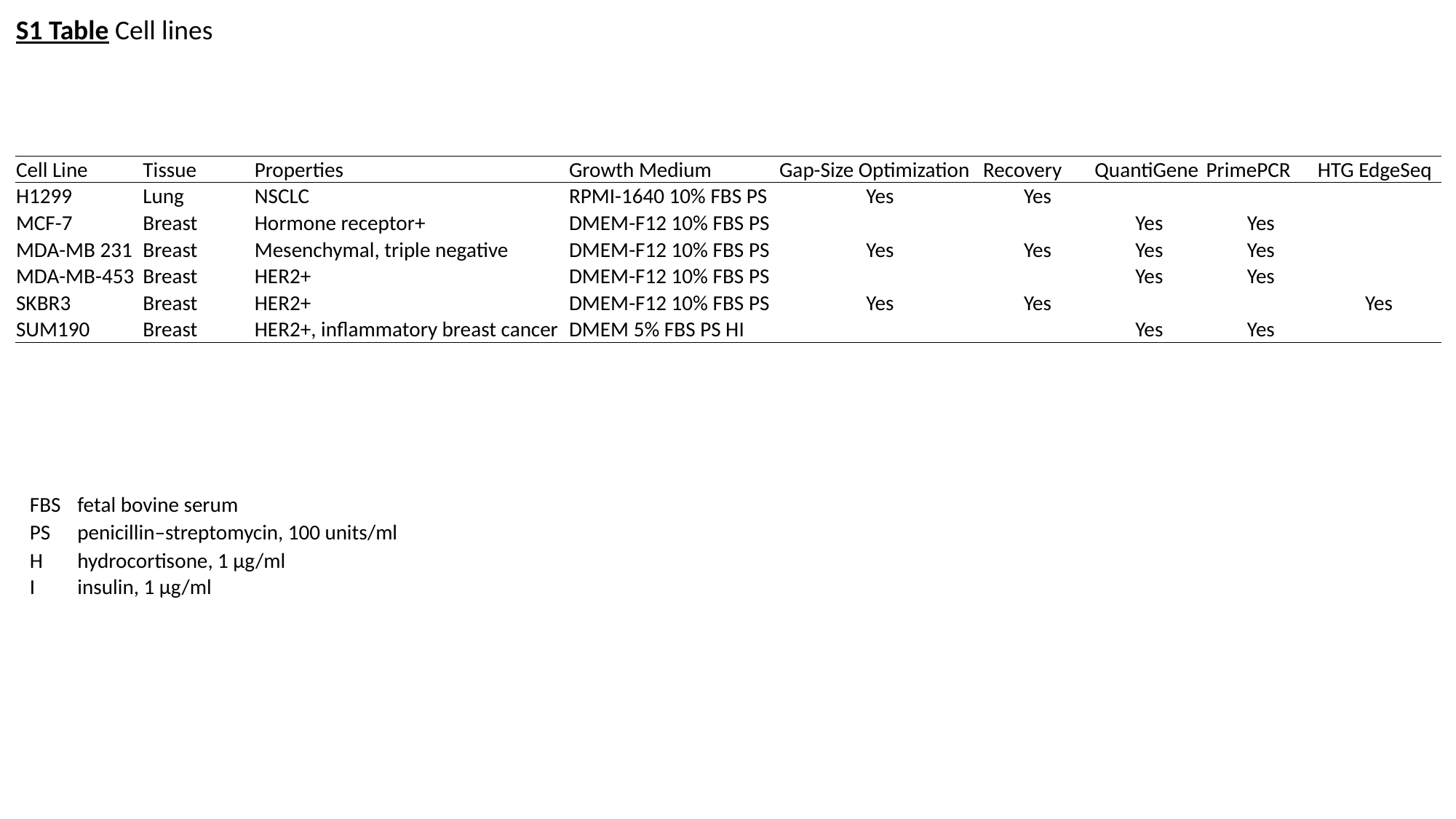

S1 Table Cell lines
| Cell Line | Tissue | Properties | Growth Medium | Gap-Size Optimization | Recovery | QuantiGene | PrimePCR | HTG EdgeSeq |
| --- | --- | --- | --- | --- | --- | --- | --- | --- |
| H1299 | Lung | NSCLC | RPMI-1640 10% FBS PS | Yes | Yes | | | |
| MCF-7 | Breast | Hormone receptor+ | DMEM-F12 10% FBS PS | | | Yes | Yes | |
| MDA-MB 231 | Breast | Mesenchymal, triple negative | DMEM-F12 10% FBS PS | Yes | Yes | Yes | Yes | |
| MDA-MB-453 | Breast | HER2+ | DMEM-F12 10% FBS PS | | | Yes | Yes | |
| SKBR3 | Breast | HER2+ | DMEM-F12 10% FBS PS | Yes | Yes | | | Yes |
| SUM190 | Breast | HER2+, inflammatory breast cancer | DMEM 5% FBS PS HI | | | Yes | Yes | |
| FBS | fetal bovine serum |
| --- | --- |
| PS | penicillin–streptomycin, 100 units/ml |
| H | hydrocortisone, 1 µg/ml |
| I | insulin, 1 µg/ml |

## Slide 2
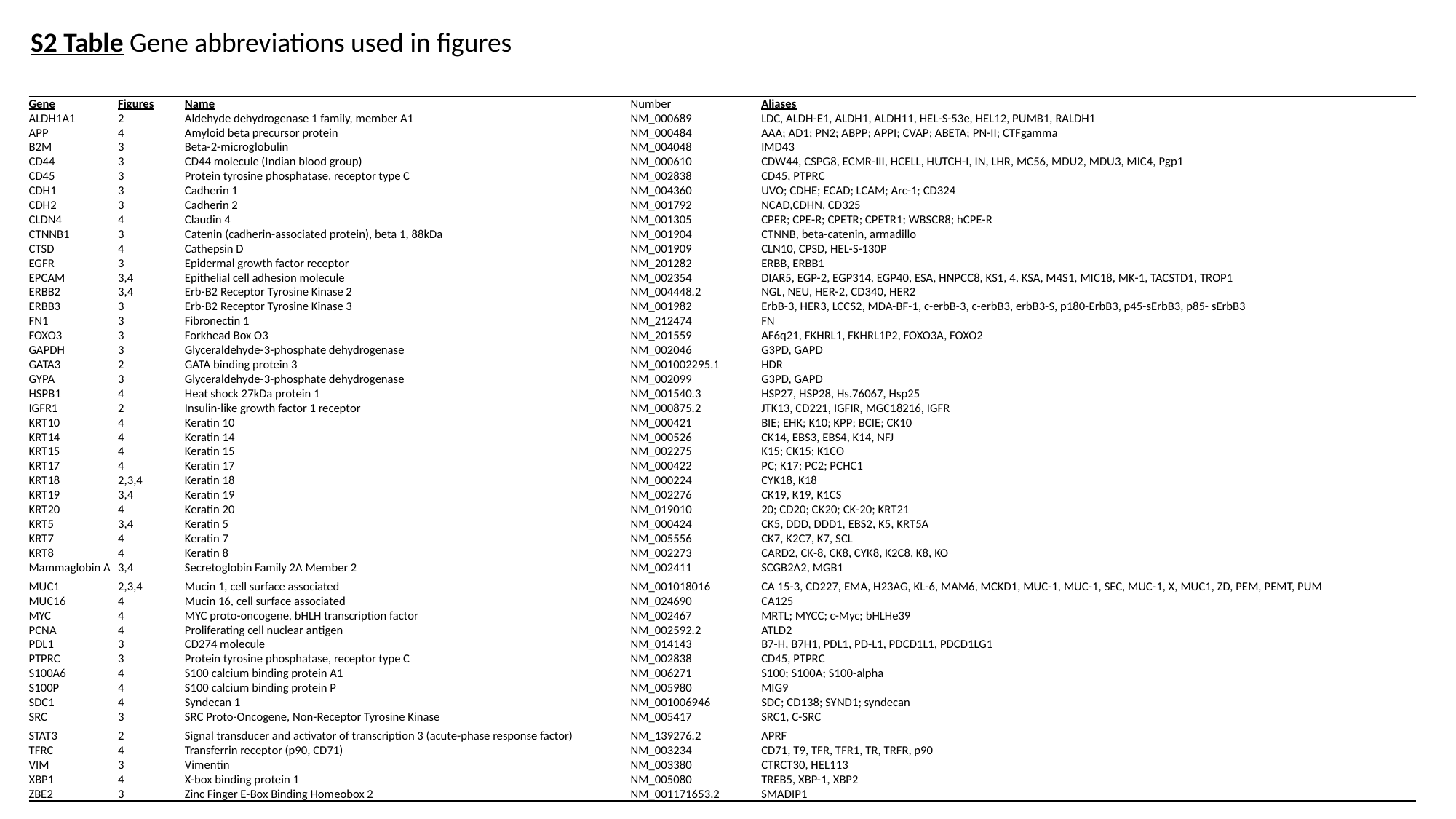

S2 Table Gene abbreviations used in figures
| Gene | Figures | Name | Number | Aliases |
| --- | --- | --- | --- | --- |
| ALDH1A1 | 2 | Aldehyde dehydrogenase 1 family, member A1 | NM\_000689 | LDC, ALDH-E1, ALDH1, ALDH11, HEL-S-53e, HEL12, PUMB1, RALDH1 |
| APP | 4 | Amyloid beta precursor protein | NM\_000484 | AAA; AD1; PN2; ABPP; APPI; CVAP; ABETA; PN-II; CTFgamma |
| B2M | 3 | Beta-2-microglobulin | NM\_004048 | IMD43 |
| CD44 | 3 | CD44 molecule (Indian blood group) | NM\_000610 | CDW44, CSPG8, ECMR-III, HCELL, HUTCH-I, IN, LHR, MC56, MDU2, MDU3, MIC4, Pgp1 |
| CD45 | 3 | Protein tyrosine phosphatase, receptor type C | NM\_002838 | CD45, PTPRC |
| CDH1 | 3 | Cadherin 1 | NM\_004360 | UVO; CDHE; ECAD; LCAM; Arc-1; CD324 |
| CDH2 | 3 | Cadherin 2 | NM\_001792 | NCAD,CDHN, CD325 |
| CLDN4 | 4 | Claudin 4 | NM\_001305 | CPER; CPE-R; CPETR; CPETR1; WBSCR8; hCPE-R |
| CTNNB1 | 3 | Catenin (cadherin-associated protein), beta 1, 88kDa | NM\_001904 | CTNNB, beta-catenin, armadillo |
| CTSD | 4 | Cathepsin D | NM\_001909 | CLN10, CPSD, HEL-S-130P |
| EGFR | 3 | Epidermal growth factor receptor | NM\_201282 | ERBB, ERBB1 |
| EPCAM | 3,4 | Epithelial cell adhesion molecule | NM\_002354 | DIAR5, EGP-2, EGP314, EGP40, ESA, HNPCC8, KS1, 4, KSA, M4S1, MIC18, MK-1, TACSTD1, TROP1 |
| ERBB2 | 3,4 | Erb-B2 Receptor Tyrosine Kinase 2 | NM\_004448.2 | NGL, NEU, HER-2, CD340, HER2 |
| ERBB3 | 3 | Erb-B2 Receptor Tyrosine Kinase 3 | NM\_001982 | ErbB-3, HER3, LCCS2, MDA-BF-1, c-erbB-3, c-erbB3, erbB3-S, p180-ErbB3, p45-sErbB3, p85- sErbB3 |
| FN1 | 3 | Fibronectin 1 | NM\_212474 | FN |
| FOXO3 | 3 | Forkhead Box O3 | NM\_201559 | AF6q21, FKHRL1, FKHRL1P2, FOXO3A, FOXO2 |
| GAPDH | 3 | Glyceraldehyde-3-phosphate dehydrogenase | NM\_002046 | G3PD, GAPD |
| GATA3 | 2 | GATA binding protein 3 | NM\_001002295.1 | HDR |
| GYPA | 3 | Glyceraldehyde-3-phosphate dehydrogenase | NM\_002099 | G3PD, GAPD |
| HSPB1 | 4 | Heat shock 27kDa protein 1 | NM\_001540.3 | HSP27, HSP28, Hs.76067, Hsp25 |
| IGFR1 | 2 | Insulin-like growth factor 1 receptor | NM\_000875.2 | JTK13, CD221, IGFIR, MGC18216, IGFR |
| KRT10 | 4 | Keratin 10 | NM\_000421 | BIE; EHK; K10; KPP; BCIE; CK10 |
| KRT14 | 4 | Keratin 14 | NM\_000526 | CK14, EBS3, EBS4, K14, NFJ |
| KRT15 | 4 | Keratin 15 | NM\_002275 | K15; CK15; K1CO |
| KRT17 | 4 | Keratin 17 | NM\_000422 | PC; K17; PC2; PCHC1 |
| KRT18 | 2,3,4 | Keratin 18 | NM\_000224 | CYK18, K18 |
| KRT19 | 3,4 | Keratin 19 | NM\_002276 | CK19, K19, K1CS |
| KRT20 | 4 | Keratin 20 | NM\_019010 | 20; CD20; CK20; CK-20; KRT21 |
| KRT5 | 3,4 | Keratin 5 | NM\_000424 | CK5, DDD, DDD1, EBS2, K5, KRT5A |
| KRT7 | 4 | Keratin 7 | NM\_005556 | CK7, K2C7, K7, SCL |
| KRT8 | 4 | Keratin 8 | NM\_002273 | CARD2, CK-8, CK8, CYK8, K2C8, K8, KO |
| Mammaglobin A | 3,4 | Secretoglobin Family 2A Member 2 | NM\_002411 | SCGB2A2, MGB1 |
| MUC1 | 2,3,4 | Mucin 1, cell surface associated | NM\_001018016 | CA 15-3, CD227, EMA, H23AG, KL-6, MAM6, MCKD1, MUC-1, MUC-1, SEC, MUC-1, X, MUC1, ZD, PEM, PEMT, PUM |
| MUC16 | 4 | Mucin 16, cell surface associated | NM\_024690 | CA125 |
| MYC | 4 | MYC proto-oncogene, bHLH transcription factor | NM\_002467 | MRTL; MYCC; c-Myc; bHLHe39 |
| PCNA | 4 | Proliferating cell nuclear antigen | NM\_002592.2 | ATLD2 |
| PDL1 | 3 | CD274 molecule | NM\_014143 | B7-H, B7H1, PDL1, PD-L1, PDCD1L1, PDCD1LG1 |
| PTPRC | 3 | Protein tyrosine phosphatase, receptor type C | NM\_002838 | CD45, PTPRC |
| S100A6 | 4 | S100 calcium binding protein A1 | NM\_006271 | S100; S100A; S100-alpha |
| S100P | 4 | S100 calcium binding protein P | NM\_005980 | MIG9 |
| SDC1 | 4 | Syndecan 1 | NM\_001006946 | SDC; CD138; SYND1; syndecan |
| SRC | 3 | SRC Proto-Oncogene, Non-Receptor Tyrosine Kinase | NM\_005417 | SRC1, C-SRC |
| STAT3 | 2 | Signal transducer and activator of transcription 3 (acute-phase response factor) | NM\_139276.2 | APRF |
| TFRC | 4 | Transferrin receptor (p90, CD71) | NM\_003234 | CD71, T9, TFR, TFR1, TR, TRFR, p90 |
| VIM | 3 | Vimentin | NM\_003380 | CTRCT30, HEL113 |
| XBP1 | 4 | X-box binding protein 1 | NM\_005080 | TREB5, XBP-1, XBP2 |
| ZBE2 | 3 | Zinc Finger E-Box Binding Homeobox 2 | NM\_001171653.2 | SMADIP1 |

## Slide 3
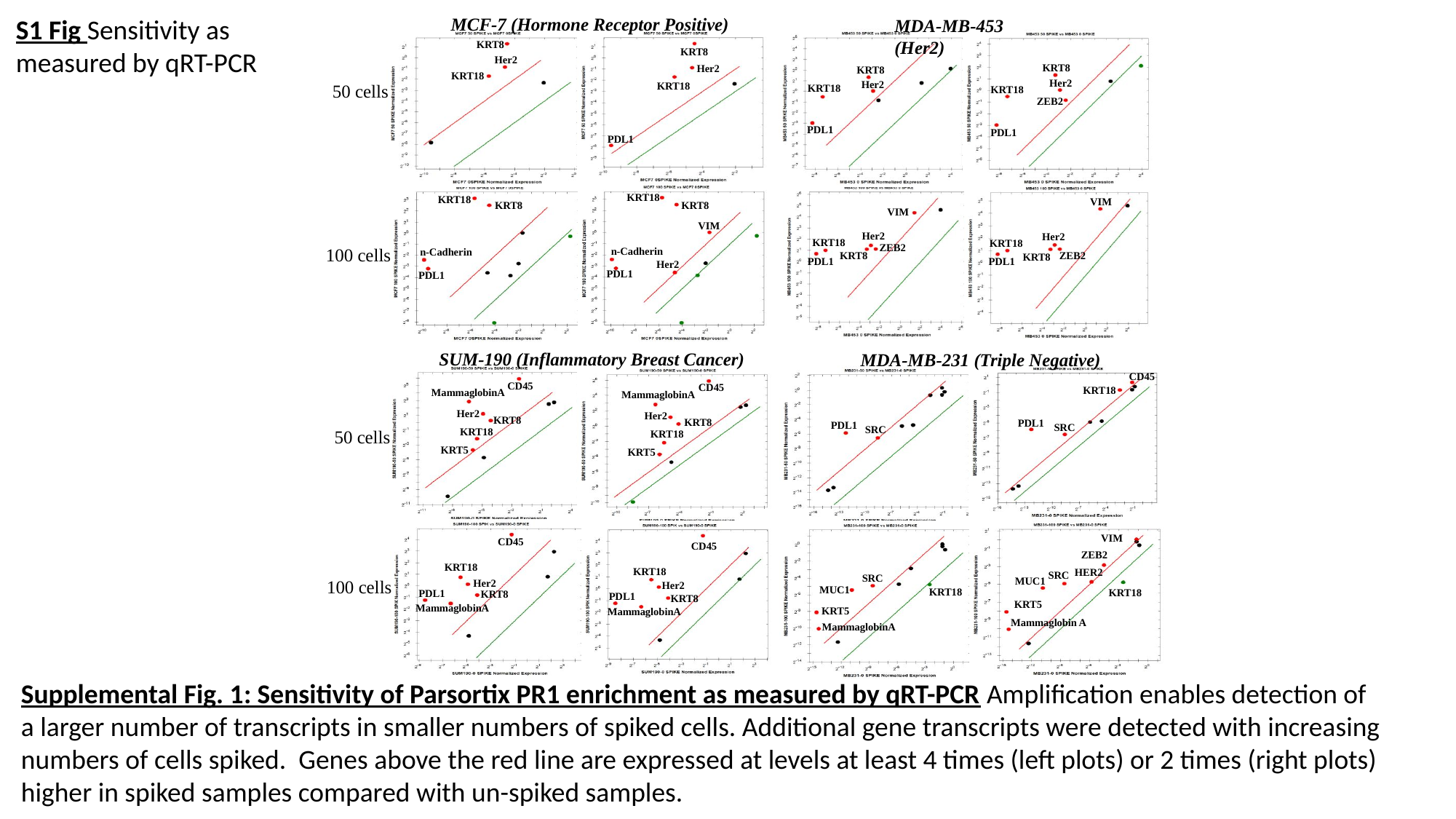

S1 Fig Sensitivity as measured by qRT-PCR
MCF-7 (Hormone Receptor Positive)
MDA-MB-453 (Her2)
KRT8
Her2
KRT18
PDL1
KRT8
Her2
KRT18
PDL1
KRT8
Her2
KRT18
KRT8
Her2
KRT18
ZEB2
PDL1
50 cells
KRT18
KRT8
VIM
n-Cadherin
Her2
PDL1
VIM
Her2
KRT18
ZEB2
KRT8
PDL1
KRT18
KRT8
n-Cadherin
PDL1
VIM
Her2
KRT18
ZEB2
KRT8
PDL1
100 cells
SUM-190 (Inflammatory Breast Cancer)
MDA-MB-231 (Triple Negative)
CD45
MammaglobinA
Her2
KRT8
KRT18
KRT5
CD45
KRT18
PDL1
SRC
PDL1
SRC
CD45
MammaglobinA
Her2
KRT8
KRT18
KRT5
50 cells
CD45
KRT18
Her2
PDL1
KRT8
MammaglobinA
VIM
VIM
ZEB2
ZEB2
HER2
SRC
MUC1
Her2
SRC
KRT18
MUC1
KRT5
Mammaglobin A
KRT5
MammaglobinA
SRC
MUC1
KRT18
KRT5
MammaglobinA
CD45
KRT18
Her2
PDL1
KRT8
MammaglobinA
100 cells
Supplemental Fig. 1: Sensitivity of Parsortix PR1 enrichment as measured by qRT-PCR Amplification enables detection of a larger number of transcripts in smaller numbers of spiked cells. Additional gene transcripts were detected with increasing numbers of cells spiked. Genes above the red line are expressed at levels at least 4 times (left plots) or 2 times (right plots) higher in spiked samples compared with un-spiked samples.

## Slide 4
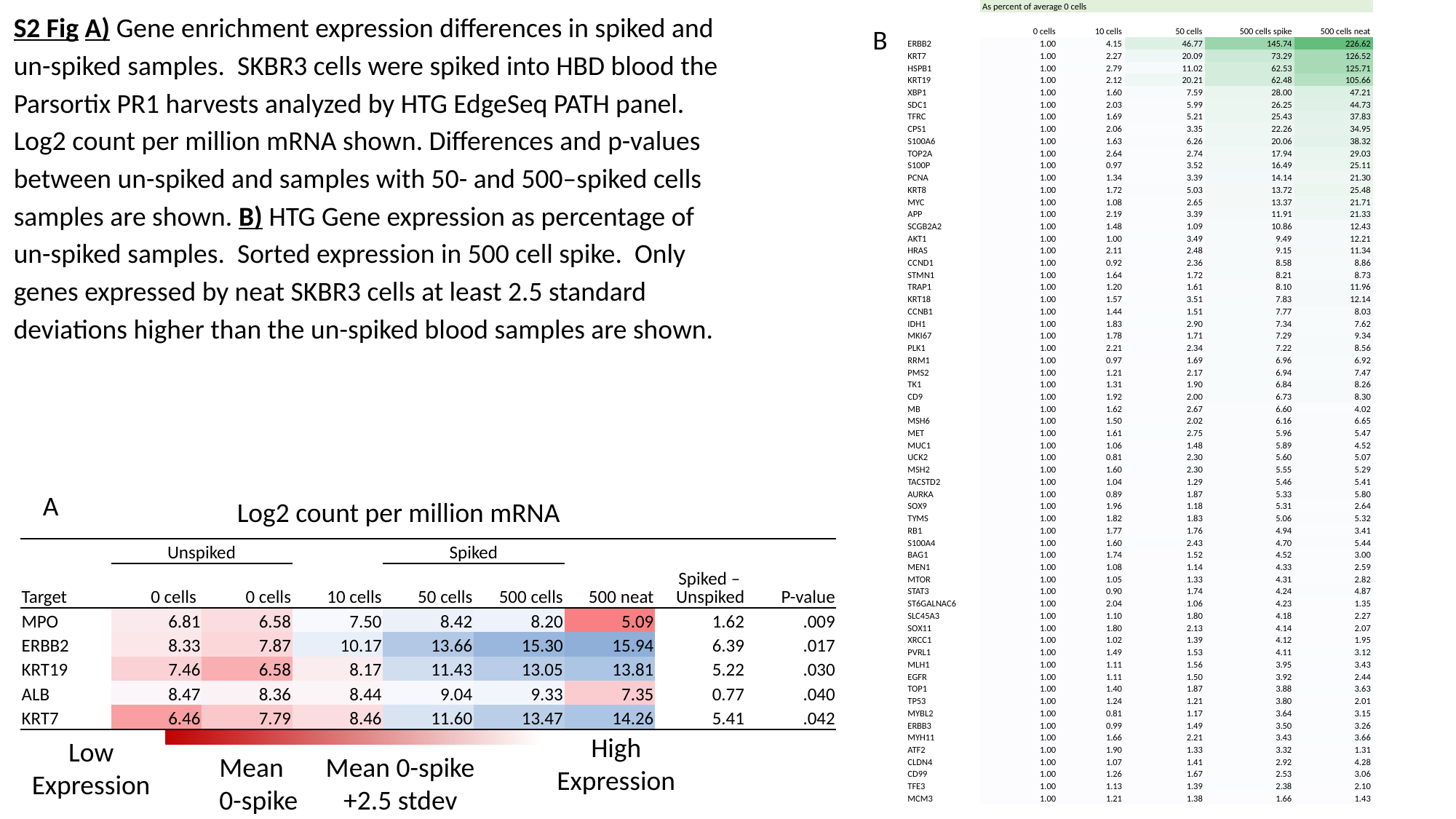

S2 Fig A) Gene enrichment expression differences in spiked and un-spiked samples. SKBR3 cells were spiked into HBD blood the Parsortix PR1 harvests analyzed by HTG EdgeSeq PATH panel. Log2 count per million mRNA shown. Differences and p-values between un-spiked and samples with 50- and 500–spiked cells samples are shown. B) HTG Gene expression as percentage of un-spiked samples. Sorted expression in 500 cell spike. Only genes expressed by neat SKBR3 cells at least 2.5 standard deviations higher than the un-spiked blood samples are shown.
| | As percent of average 0 cells | | | | |
| --- | --- | --- | --- | --- | --- |
| | 0 cells | 10 cells | 50 cells | 500 cells spike | 500 cells neat |
| ERBB2 | 1.00 | 4.15 | 46.77 | 145.74 | 226.62 |
| KRT7 | 1.00 | 2.27 | 20.09 | 73.29 | 126.52 |
| HSPB1 | 1.00 | 2.79 | 11.02 | 62.53 | 125.71 |
| KRT19 | 1.00 | 2.12 | 20.21 | 62.48 | 105.66 |
| XBP1 | 1.00 | 1.60 | 7.59 | 28.00 | 47.21 |
| SDC1 | 1.00 | 2.03 | 5.99 | 26.25 | 44.73 |
| TFRC | 1.00 | 1.69 | 5.21 | 25.43 | 37.83 |
| CPS1 | 1.00 | 2.06 | 3.35 | 22.26 | 34.95 |
| S100A6 | 1.00 | 1.63 | 6.26 | 20.06 | 38.32 |
| TOP2A | 1.00 | 2.64 | 2.74 | 17.94 | 29.03 |
| S100P | 1.00 | 0.97 | 3.52 | 16.49 | 25.11 |
| PCNA | 1.00 | 1.34 | 3.39 | 14.14 | 21.30 |
| KRT8 | 1.00 | 1.72 | 5.03 | 13.72 | 25.48 |
| MYC | 1.00 | 1.08 | 2.65 | 13.37 | 21.71 |
| APP | 1.00 | 2.19 | 3.39 | 11.91 | 21.33 |
| SCGB2A2 | 1.00 | 1.48 | 1.09 | 10.86 | 12.43 |
| AKT1 | 1.00 | 1.00 | 3.49 | 9.49 | 12.21 |
| HRAS | 1.00 | 2.11 | 2.48 | 9.15 | 11.34 |
| CCND1 | 1.00 | 0.92 | 2.36 | 8.58 | 8.86 |
| STMN1 | 1.00 | 1.64 | 1.72 | 8.21 | 8.73 |
| TRAP1 | 1.00 | 1.20 | 1.61 | 8.10 | 11.96 |
| KRT18 | 1.00 | 1.57 | 3.51 | 7.83 | 12.14 |
| CCNB1 | 1.00 | 1.44 | 1.51 | 7.77 | 8.03 |
| IDH1 | 1.00 | 1.83 | 2.90 | 7.34 | 7.62 |
| MKI67 | 1.00 | 1.78 | 1.71 | 7.29 | 9.34 |
| PLK1 | 1.00 | 2.21 | 2.34 | 7.22 | 8.56 |
| RRM1 | 1.00 | 0.97 | 1.69 | 6.96 | 6.92 |
| PMS2 | 1.00 | 1.21 | 2.17 | 6.94 | 7.47 |
| TK1 | 1.00 | 1.31 | 1.90 | 6.84 | 8.26 |
| CD9 | 1.00 | 1.92 | 2.00 | 6.73 | 8.30 |
| MB | 1.00 | 1.62 | 2.67 | 6.60 | 4.02 |
| MSH6 | 1.00 | 1.50 | 2.02 | 6.16 | 6.65 |
| MET | 1.00 | 1.61 | 2.75 | 5.96 | 5.47 |
| MUC1 | 1.00 | 1.06 | 1.48 | 5.89 | 4.52 |
| UCK2 | 1.00 | 0.81 | 2.30 | 5.60 | 5.07 |
| MSH2 | 1.00 | 1.60 | 2.30 | 5.55 | 5.29 |
| TACSTD2 | 1.00 | 1.04 | 1.29 | 5.46 | 5.41 |
| AURKA | 1.00 | 0.89 | 1.87 | 5.33 | 5.80 |
| SOX9 | 1.00 | 1.96 | 1.18 | 5.31 | 2.64 |
| TYMS | 1.00 | 1.82 | 1.83 | 5.06 | 5.32 |
| RB1 | 1.00 | 1.77 | 1.76 | 4.94 | 3.41 |
| S100A4 | 1.00 | 1.60 | 2.43 | 4.70 | 5.44 |
| BAG1 | 1.00 | 1.74 | 1.52 | 4.52 | 3.00 |
| MEN1 | 1.00 | 1.08 | 1.14 | 4.33 | 2.59 |
| MTOR | 1.00 | 1.05 | 1.33 | 4.31 | 2.82 |
| STAT3 | 1.00 | 0.90 | 1.74 | 4.24 | 4.87 |
| ST6GALNAC6 | 1.00 | 2.04 | 1.06 | 4.23 | 1.35 |
| SLC45A3 | 1.00 | 1.10 | 1.80 | 4.18 | 2.27 |
| SOX11 | 1.00 | 1.80 | 2.13 | 4.14 | 2.07 |
| XRCC1 | 1.00 | 1.02 | 1.39 | 4.12 | 1.95 |
| PVRL1 | 1.00 | 1.49 | 1.53 | 4.11 | 3.12 |
| MLH1 | 1.00 | 1.11 | 1.56 | 3.95 | 3.43 |
| EGFR | 1.00 | 1.11 | 1.50 | 3.92 | 2.44 |
| TOP1 | 1.00 | 1.40 | 1.87 | 3.88 | 3.63 |
| TP53 | 1.00 | 1.24 | 1.21 | 3.80 | 2.01 |
| MYBL2 | 1.00 | 0.81 | 1.17 | 3.64 | 3.15 |
| ERBB3 | 1.00 | 0.99 | 1.49 | 3.50 | 3.26 |
| MYH11 | 1.00 | 1.66 | 2.21 | 3.43 | 3.66 |
| ATF2 | 1.00 | 1.90 | 1.33 | 3.32 | 1.31 |
| CLDN4 | 1.00 | 1.07 | 1.41 | 2.92 | 4.28 |
| CD99 | 1.00 | 1.26 | 1.67 | 2.53 | 3.06 |
| TFE3 | 1.00 | 1.13 | 1.39 | 2.38 | 2.10 |
| MCM3 | 1.00 | 1.21 | 1.38 | 1.66 | 1.43 |
B
A
Log2 count per million mRNA
| | Unspiked | | | Spiked | | | Spiked – Unspiked | |
| --- | --- | --- | --- | --- | --- | --- | --- | --- |
| Target | 0 cells | 0 cells | 10 cells | 50 cells | 500 cells | 500 neat | | P-value |
| MPO | 6.81 | 6.58 | 7.50 | 8.42 | 8.20 | 5.09 | 1.62 | .009 |
| ERBB2 | 8.33 | 7.87 | 10.17 | 13.66 | 15.30 | 15.94 | 6.39 | .017 |
| KRT19 | 7.46 | 6.58 | 8.17 | 11.43 | 13.05 | 13.81 | 5.22 | .030 |
| ALB | 8.47 | 8.36 | 8.44 | 9.04 | 9.33 | 7.35 | 0.77 | .040 |
| KRT7 | 6.46 | 7.79 | 8.46 | 11.60 | 13.47 | 14.26 | 5.41 | .042 |
High
Expression
Low
Expression
Mean
0-spike
Mean 0-spike
+2.5 stdev

## Slide 5
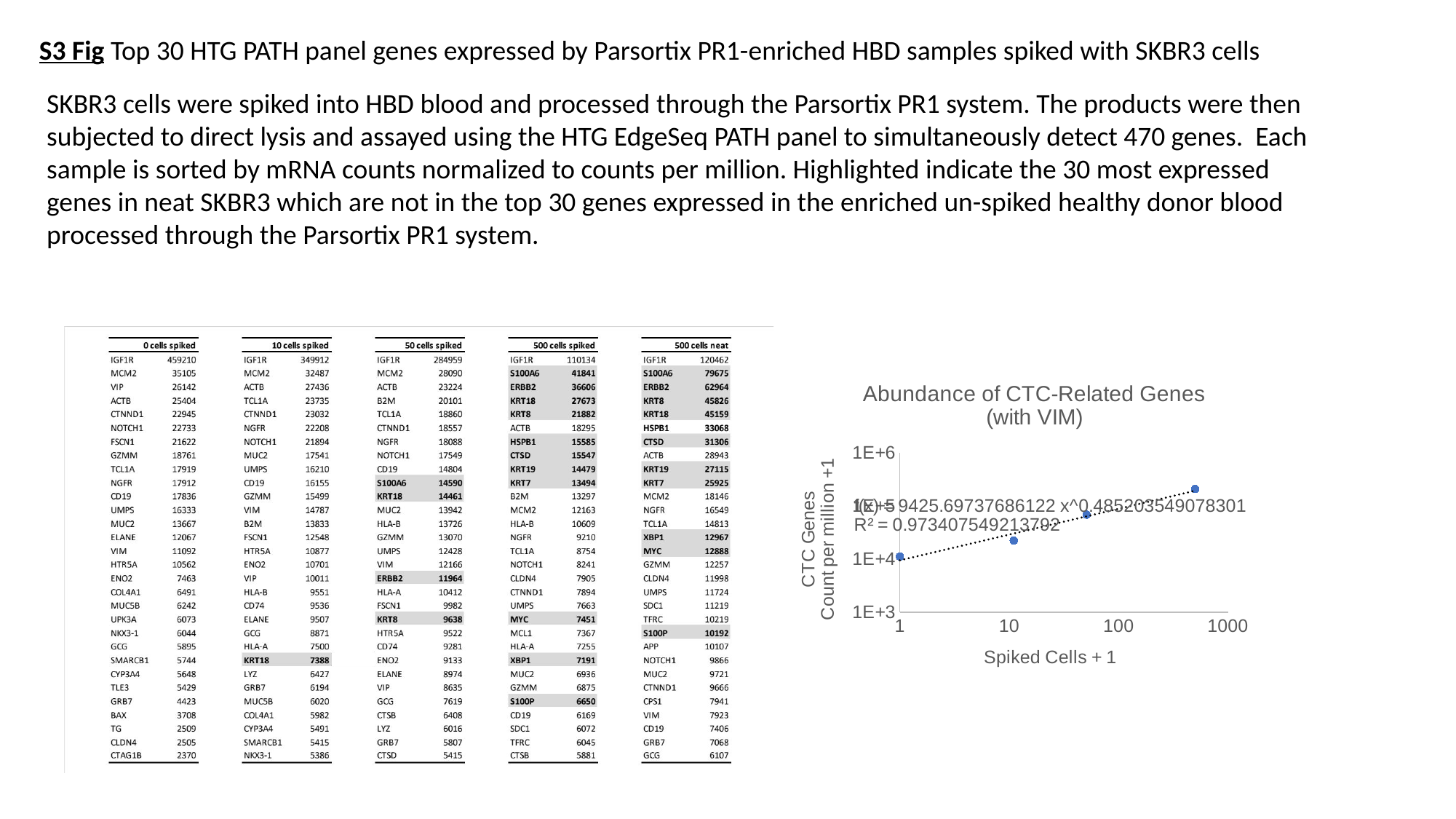

S3 Fig Top 30 HTG PATH panel genes expressed by Parsortix PR1-enriched HBD samples spiked with SKBR3 cells
SKBR3 cells were spiked into HBD blood and processed through the Parsortix PR1 system. The products were then subjected to direct lysis and assayed using the HTG EdgeSeq PATH panel to simultaneously detect 470 genes. Each sample is sorted by mRNA counts normalized to counts per million. Highlighted indicate the 30 most expressed genes in neat SKBR3 which are not in the top 30 genes expressed in the enriched un-spiked healthy donor blood processed through the Parsortix PR1 system.
### Chart: Abundance of CTC-Related Genes
(with VIM)
| Category | |
|---|---|

## Slide 6
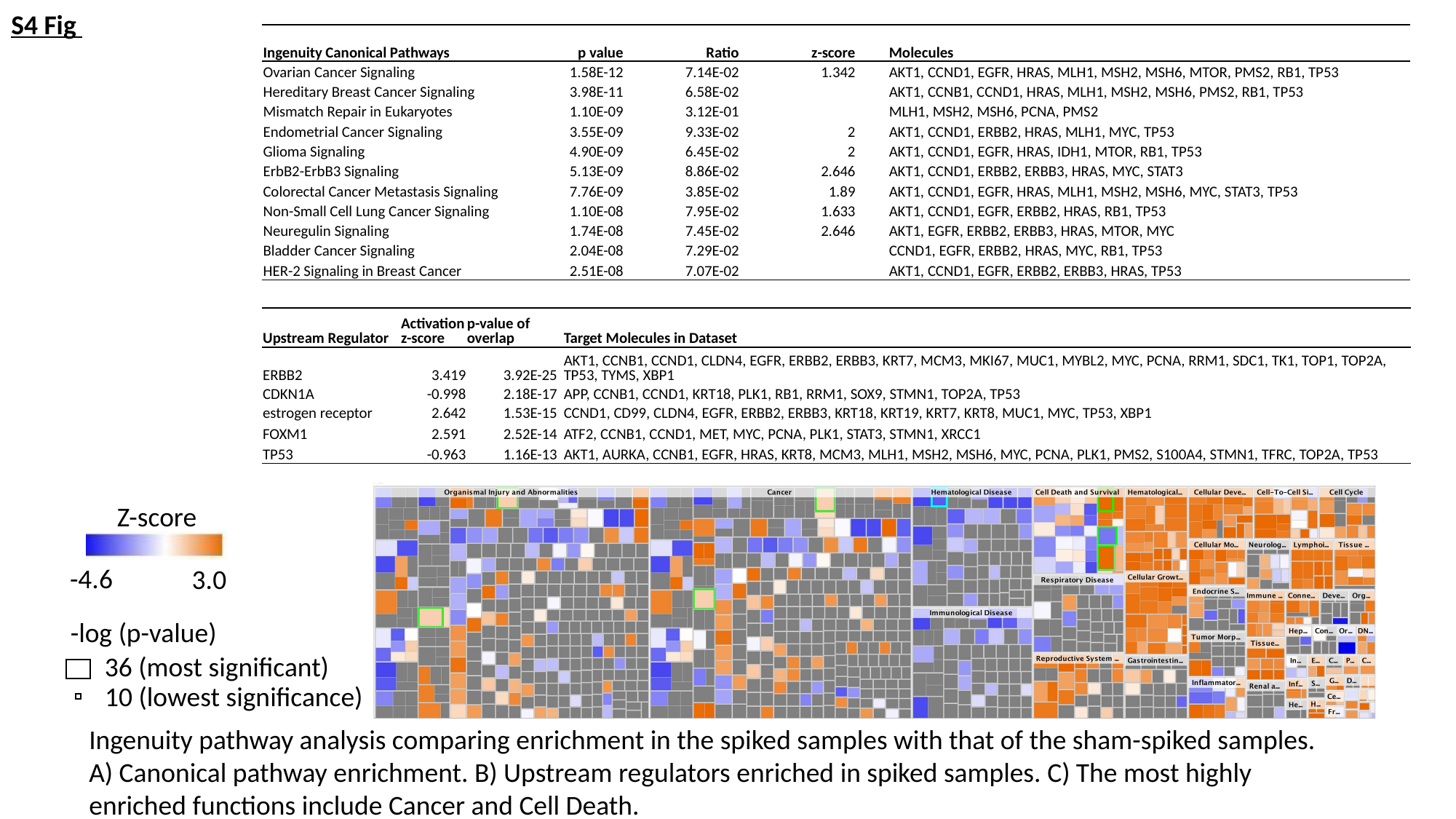

S4 Fig
| Ingenuity Canonical Pathways | p value | Ratio | z-score | | Molecules |
| --- | --- | --- | --- | --- | --- |
| Ovarian Cancer Signaling | 1.58E-12 | 7.14E-02 | 1.342 | | AKT1, CCND1, EGFR, HRAS, MLH1, MSH2, MSH6, MTOR, PMS2, RB1, TP53 |
| Hereditary Breast Cancer Signaling | 3.98E-11 | 6.58E-02 | | | AKT1, CCNB1, CCND1, HRAS, MLH1, MSH2, MSH6, PMS2, RB1, TP53 |
| Mismatch Repair in Eukaryotes | 1.10E-09 | 3.12E-01 | | | MLH1, MSH2, MSH6, PCNA, PMS2 |
| Endometrial Cancer Signaling | 3.55E-09 | 9.33E-02 | 2 | | AKT1, CCND1, ERBB2, HRAS, MLH1, MYC, TP53 |
| Glioma Signaling | 4.90E-09 | 6.45E-02 | 2 | | AKT1, CCND1, EGFR, HRAS, IDH1, MTOR, RB1, TP53 |
| ErbB2-ErbB3 Signaling | 5.13E-09 | 8.86E-02 | 2.646 | | AKT1, CCND1, ERBB2, ERBB3, HRAS, MYC, STAT3 |
| Colorectal Cancer Metastasis Signaling | 7.76E-09 | 3.85E-02 | 1.89 | | AKT1, CCND1, EGFR, HRAS, MLH1, MSH2, MSH6, MYC, STAT3, TP53 |
| Non-Small Cell Lung Cancer Signaling | 1.10E-08 | 7.95E-02 | 1.633 | | AKT1, CCND1, EGFR, ERBB2, HRAS, RB1, TP53 |
| Neuregulin Signaling | 1.74E-08 | 7.45E-02 | 2.646 | | AKT1, EGFR, ERBB2, ERBB3, HRAS, MTOR, MYC |
| Bladder Cancer Signaling | 2.04E-08 | 7.29E-02 | | | CCND1, EGFR, ERBB2, HRAS, MYC, RB1, TP53 |
| HER-2 Signaling in Breast Cancer | 2.51E-08 | 7.07E-02 | | | AKT1, CCND1, EGFR, ERBB2, ERBB3, HRAS, TP53 |
| Upstream Regulator | Activation z-score | p-value of overlap | | Target Molecules in Dataset |
| --- | --- | --- | --- | --- |
| ERBB2 | 3.419 | 3.92E-25 | | AKT1, CCNB1, CCND1, CLDN4, EGFR, ERBB2, ERBB3, KRT7, MCM3, MKI67, MUC1, MYBL2, MYC, PCNA, RRM1, SDC1, TK1, TOP1, TOP2A, TP53, TYMS, XBP1 |
| CDKN1A | -0.998 | 2.18E-17 | | APP, CCNB1, CCND1, KRT18, PLK1, RB1, RRM1, SOX9, STMN1, TOP2A, TP53 |
| estrogen receptor | 2.642 | 1.53E-15 | | CCND1, CD99, CLDN4, EGFR, ERBB2, ERBB3, KRT18, KRT19, KRT7, KRT8, MUC1, MYC, TP53, XBP1 |
| FOXM1 | 2.591 | 2.52E-14 | | ATF2, CCNB1, CCND1, MET, MYC, PCNA, PLK1, STAT3, STMN1, XRCC1 |
| TP53 | -0.963 | 1.16E-13 | | AKT1, AURKA, CCNB1, EGFR, HRAS, KRT8, MCM3, MLH1, MSH2, MSH6, MYC, PCNA, PLK1, PMS2, S100A4, STMN1, TFRC, TOP2A, TP53 |
Z-score
-4.6
3.0
-log (p-value)
36 (most significant)
10 (lowest significance)
Ingenuity pathway analysis comparing enrichment in the spiked samples with that of the sham-spiked samples. A) Canonical pathway enrichment. B) Upstream regulators enriched in spiked samples. C) The most highly enriched functions include Cancer and Cell Death.
