# Supplementary material for: Antigen-agnostic microfluidics-based circulating tumor cell enrichment and downstream molecular characterization
Source: PLoS One. 2020 Oct 23;15(10):e0241123. doi: 10.1371/journal.pone.0241123 (PMC7584183; doi:10.1371/journal.pone.0241123)
Supplement: Attachment — Submitted filename: Response to Reviewers.pdf [file pone.0241123.s002.pdf]

THE UNIVERSITY OF TEXAS
MD Anderson ~~Cancer~~ Center
Making Cancer History®

Evan N. Cohen, PhD
Instructor
encohen@mdanderson.org
Department of Hematopathology
Pathology and Laboratory Medicine
T 713-745-2132
Unit 0054
1515 Holcombe Blvd
Houston, Texas 77030

August 18, 2020

Academic Reviewers and Editorial Board of PLOS ONE

Dear Reviewers and Dr. Chalmers:

It is my pleasure to submit for your review our revised manuscript "Antigen-agnostic microfluidics-based circulating tumor cell enrichment and downstream molecular characterization." We thank the reviewer and Dr. Chalmers for the comments. Please see below for an accounting of each comment.

Journal Requirements:

1. Style Requirements
   a. Will be finalized after peer review if that is acceptable
2. Ethics statement
   a. Revised
3. "data not shown"
   a. Removed
4. financial disclosure
   a. Amended as suggested
5. Competing Interests
   a. I have read the journal's policy and the authors of this manuscript have the following competing interests: JMR serves on the scientific advisory board for Angle PLC. This does not alter our adherence to PLOS ONE policies on sharing data and materials.
6. Abstract Publication
   a. Data was partially presented as a poster in the American Association for Clinical Chemistry Annual Conference 2016, Philadelphia PA. It was neither peer-reviewed nor formally published.
7. Figure copyright
   a. There are no copyrighted figures in the manuscript, all figures were prepared by the authors. We have added a citation in the Methods section for the Ingenuity Pathway Analysis shown in supplemental figure 4.

Reviewer Comments to the Author:

1. Deformation and capillary flow required by the platform should be expected to result in lysis of some patient CTCs. How are these phenomena represented in the recovery data? is this the source of spiked cell loss?
   a. We agree, but quantifying this effect was beyond the scope of this project. We added a statement in the discussion.
2. Gap Size. Please expand on the concept of gap size. why is there a decreased recovery with increased gap size?
   a. Added to discussion, second paragraph. Any population of cells has a distribution of cell sizes, any cells smaller than the gap size will pass through the system without enrichment. A larger portion of this distribution is lost to capture as the gap size threshold is increased.
3. Hybridization Linearity Data: for the hybridization data, I read the manuscript as saying 4 spiked specimens assayed in duplicate for each cell line to generate the data. is that correct? Can the recovery data for all experiments be pooled to generate a recovery regression model and equation related to spike level?
   a. Included below is a single regression for KRT18 pooling all data using cell line as a factor, the manuscript shows separate regressions for each cell line. Only technical duplicates are available for the spiked cells, but the reviewer is otherwise correct, the un-spiked samples have 4 biological replicates. As seen in the model below, expression is significantly related to the number of cells spiked ($p = 0.000171$). We changed the recovery regression R^2 values in the manuscript (line 323 tracked version, line 308 clean) to include the replicate points as suggested by the reviewer here, reflecting both linearity and precision. The higher R^2 shown before only reflected linearity.

Recovery regression model

KRT18	Estimate	Std. Error	t value	Pr(>|t|)
(Intercept)	78.0053	422.7937	0.184	0.855
# Cells Spiked	0.4174	0.0958	4.357	0.000171
Cell Line MCF7	control			
Cell Line MDA231	-629	567.602	-1.108	0.277562
Cell Line MDA453	-636.25	567.602	-1.121	0.272183
Cell Line SUM190	946.9375	567.602	1.668	0.106812

Multiple R-squared: 0.521, Adjusted R-squared: 0.45
Model: lm(KRT18 ~ Cells Spiked + Cell Line)

   b. related to this, figure 1b shows that recovery is cell line dependent. is recovery cell size dependent?
      Yes. Recovery decreases for smaller cells.

4. In figure 3, are the spiked cell numbers per 5 mL or 1 mL of blood, please state. Also please specify the elapsed time between harvest, spiking and purification.
   a. The number of spiked cells is reported per sample of blood enriched by the system, generally at least 5mL. *Added to methods and figure 3 legend.*
   b. Typically start processing within 4 hours of phlebotomy. Added typical times to methods section.
5. What is the optimal method of cell preservation prior to qRT-PCR analysis?
   a. Good question! Immediate lysis is the best solution we have found. We noted that single shot buffers that take the sample directly to PCR without nucleic acid enrichment did not work well, particularly for florescent-based assays (e.g. qRT-PCR) when trace red blood cells remained in the sample. *Not added to manuscript.*
6. In figure 3C, the SKBR3 10 cell spike recovery level varied over 6 CT values. please comment.
   a. The small change in control gene expression suggests this is related to CTC recovery and not a PCR artifact. We felt it was best to include all data without censoring, irrespective of expectations. As the reviewer astutely noted above, enrichment is dependent on cell size, which varies even within a single culture and was not strictly controlled prior to enrichment. Additionally, larger cells contain more RNA. We could also speculate that user-dependent variance in micromanipulation also played a role. Such variances are exacerbated when working with small cell numbers. *Not added to manuscript.*
7. This manuscript should include data on performance of this method compared to the cell search on a small panel of human specimens.
   a. Several publications have compared CellSearch and Parsortix and the citations have been updated in the revised manuscript. Chudziak et al show that enrichment of EPCAM+ cells is similar by CellSearch and Parsortix. PMID: 26605519 DOI: 10.1039/c5an02156a and PMID: 26789903 PMCID: PMC5069649 DOI: 10.1002/ijc.30007

Thank you for your consideration of this revised manuscript.

Sincerely,

Evan N. Cohen, PhD
Instructor, Department of Hematopathology
